# Event and Comment

**Published:** 1922-09

**Authors:** 


### DENTAL REGISTER

THE MAGAZINE OF DENTISTRY

Vol. LXXVI SEPTEMBER, 1922 No. 9

### EVENT AND COMMENT

**Professional or Commercial Standards!** There now seems to be a general notion that dentists do not know much about practical business methods, and that too few practitioners make and save enough money to keep them from want when their time comes and their unproductive period is reached. For this reason the economic problems are being much discussed and studied with an interest that is somewhat irritating, to say the least. It almost seems that the benevolent professional ideals of former days are liable to be much commercialized and changed to more selfish methods, rather than to become more wholesome and ethical in practice.

If there is any probability that the practice of dentistry can not be continued on the high professional plane that has been made the basis for modern ethical dental practice, a position which we have maintained proudly as a sister of other professions and arts, it would seem that it is now high time that some serious scientific study of this problem should be made, and a definite standard be found that the true status of dental ethics may be determined.

In such a study we may come to the conclusion that it will be best for ethical standards to forego luxuries and extravagances and live simpler and more frugal lives. Such a change of ideals may not meet with favor, and present ideals will not be generally accepted. The present reign of profiteering will certainly need to be changed to make it comport with the idea of the "golden rule" system.

Can and will professional men make such a readjustment without willingly doing their part? In times past have not professional men led off in all such programs; and may we not anticipate that they will surely do their part now? Historically, doctors were first taught the science or art of healing and have always practiced it as a sacred duty. We are today more than ever before making strenuous endeavors to discover new methods of relieving human suffering and of conserving the health of the people, and we take pride in our accomplishments. Prophylaxis and hygiene seem generally to be recognized as vital health propositions, and the public approves and co-operates. We sometimes think all do not co-operate as intelligently as they should, but all do not yet believe, and some of our findings are not yet verified; however, reasonable progress is assured. Some ignorant as well as some viscious traditions will have to be eradicated before a true health program can be established. No more conspicuous hindrance to this health program is encountered than that which is found in the present widespread commercialism, which seems to block every effort to create the finer sentiments and practices for the altruistic health program. It is difficult to understand why any sane person should interfere with so worthy an ideal, and we can't understand why even some medical men can be so selfish that they stand in the way of every good cause which does not appeal to their greed for gain and vain glory. But these are matters which time will clear away, and the old and time-tested health ideals will replace present commercialism, so far as they concern medical and dental practice. In the meantime every dentist should make it his first duty to establish, for his own good, a complete health program, which will enable him to contribute his proportion of efficient service; reserving whatever may be wise and practicable from all his resources a reasonable accumulation for old age and accidental emergencies. Study to save and utilize, not to hoard.

